# Perceptions of medical faculty on an e-learning module providing faculty development

**DOI:** 10.51894/001c.168172

**Published:** 2026-08-28

**Authors:** Bethany J Figg, Troy Hicks, Sally A Santen, Clara Bihn, Steve Vance, Mary Jo Wagner

**Affiliations:** 1 Graduate Medical Education Central Michigan University; 2 Teacher and Special Education Department Central Michigan University; 3 College of Medicine University of Cincinnati; 4 College of Medicine Central Michigan University

**Keywords:** comparison study, e-learning, faculty development, thematic analysis

## Abstract

**INTRODUCTION:**

Although medical educators participate in faculty development, they do not always find this education time well spent, or helpful for improving their practice. This mixed-methods randomized comparative study aimed to compare perceptions of two faculty development modalities by comparing a traditional method of learning (text-based PDF with information) with an online learning method (e-learning module).

**METHODS:**

In December of 2021, an instructionally designed e-learning module (experimental) and a text-only PDF with the same information (control) were randomly assigned to 48 faculty via Qualtrics randomizer feature, with no investigator involvement in the assignment, in four medical specialties at a medium-sized Midwest academic medical institution. Questionnaires were distributed to all faculty, pre-exercise, to gather demographics, previous faculty development experience, and responses to a practice exercise. Faculty were then provided with their randomly assigned faculty development task to complete and a post-exercise survey to discover which modality was assigned and gather their perceptions of the usefulness of the delivery method. Six (12.5%) volunteers offered to discuss the two modalities in a focus group. 29 (60.4%) of the 48 faculty completed the pre-survey, educational exercise, and post-survey. Quantitative and qualitative data were triangulated.

**RESULTS:**

Independent samples t-tests of Likert-style survey results of faculty perceptions about the e-learning module versus the text-based PDF showed no significant difference. Thematic analysis of open-ended survey responses revealed a preference for the e-learning module when learning, and the text-based PDF for reference post-learning. Finally, a focus group revealed that providing tools through both the text-only PDF and the e-learning module was preferred over only providing one or the other.

**CONCLUSIONS:**

While the purpose of the study was to determine which modality was more useful, the statistical comparisons were exploratory and underpowered. Still, results suggest that medical educators may find this approach beneficial for other types of education when designing online faculty development.

## INTRODUCTION

Swift changes in medical knowledge, healthcare delivery systems, and instructional methods for medical education and faculty development have been a driving force to utilizing technology for teaching tools.^1^ Due to rapidly advancing biomedical research leading to major accomplishments, medical practice and health policy changes at a fast pace; therefore, physicians need to keep themselves informed to stay abreast of the continuously improving patient care.^2^

Traditionally, education in medicine has relied on textbooks, in-person workshops, classroom didactics, and question banks to acquire knowledge,^3^ but medical education has been moving their textbooks to e-books enhanced with interactive media, and computer technology has introduced e-learning modalities that can be updated instantly, transcend geographical boundaries, and accessed at any time.^4^ In 2020, the COVID-19 pandemic shifted the novel addition of e-learning in medical education to a primary source of education for many medical learners.^5^

The institution described in this study manages faculty development through various formats, including traditional face-to-face sessions and e-learning options. Although the electronic format was favored for its accessibility and lack of travel commitments, logistical challenges hindered its effectiveness, especially for community and volunteer clinical faculty. Evidence suggests that online faculty development is at least comparable to traditional training, but learner engagement and critical factors of success depend on the relevance to perceived needs and instructional objectives, effective communication, and adequate time to complete program activities.^6^

Moreover, effective feedback on resident performance is necessary for achieving milestones towards independent practice, yet residents report they receive this infrequently.^7–9^ The Accreditation Council for Graduate Medical Education (ACGME) requires faculty to directly observe, evaluate, and provide feedback (typically narrative and numerical) on resident performance during each rotation in order to facilitate opportunities for improvement in patient care as well as resident advancement and promotion.^10^ Implementing and assessing the milestones for each resident by faculty is a complex educational intervention.^11^ Some barriers in providing meaningful feedback, as reported by faculty, include time constraints, the often brief duration of relationship between preceptor and trainees, inconsistencies among evaluations, quantity of evaluations, lack of training for faculty, and typical concerns of how certain statements may affect resident progression.^12,13^

Addressing issues with feedback on the milestones noted above prompted the creation of the e-learning module. This study aimed to educate faculty on effective feedback practices to foster better habits for interacting with residents. Recognizing the importance of valuable feedback for resident growth, the researchers sought a high-impact electronic tool to educate faculty. The research question was “Which is the preferred delivery method - text-only document or e-learning module - for providing faculty development on effective feedback practices?”.

## METHODS

Faculty perceptions of e-learning interactive modules were compared to text-based learning through a survey and focus group utilizing a mixed-methods randomized comparative design.

### Description and Creation of the Educational Intervention

In order to compare faculty perceptions of e-learning for faculty development, two modalities were created to deliver identical content for the educational intervention: a text-based PDF, and an interactive e-learning module created with the Articulate^®^ Rise360 software. The text-based downloadable PDF was created by adding content to a Microsoft Word document, then converted to a PDF.

The content for the e-learning module and text-based PDF was copied or closely derived from training documents provided by the ACGME. The ACGME Milestones for each specialty were pulled from the latest milestone update implemented on July 1, 2021. Both modalities contained the same educational material, including the purpose of the milestones (to give residents the markers they need to reach competency), an introduction to a feedback tool with validity evidence, Hattie and Timperley’s Model for Feedback (to give the residents feedback in three parts: 1. Where am I going? (feeding up), 2. How am I going? (feeding back), and 3. Where to next? (feeding forward)).^14,15^

Utilizing the guidelines provided by Clark and Mayer,^16^ the e-learning module was crafted to address principles of multimedia design, contiguity, redundancy, coherence, personalization, and segmenting. These were applied with graphics, interactive components, reactive responses, and removal of extraneous materials through pilot testing.

The Articulate^®^ Rise 360 program utilizes the latest web technologies and automatically adapts to any type of device through responsive authoring.^17^ A drawback to utilizing Articulate® Rise 360 is that the subscription-based service will delete any modules created and any data collected once the subscription has ended.

The e-learning module did not include any additional learning materials that did not appear in the text-based PDF, but did provide additional opportunities for interactivity, such as assessment of the knowledge that participants had gained. A link to the PDF and screen view from the e-learning module can be found in Table 1.

**Table 1.**
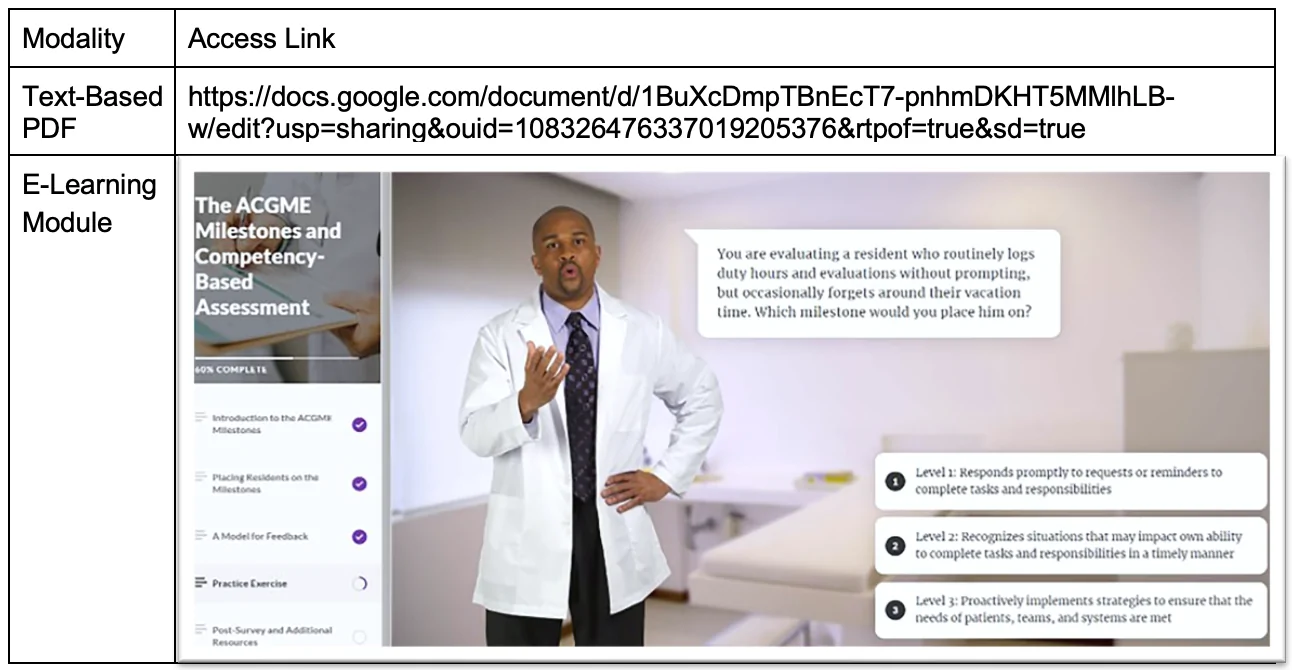
Educational Intervention

The text-based PDF and the e-learning module were pilot tested by four physicians – two inside the lead author’s organization and two outside. Two were emergency medicine physicians, one was a general surgeon, and one was a cardiologist. Each physician provided feedback that was incorporated into the educational interventions to improve the flow, instructions, and content before delivering them to the groups.

### Implementation of the Educational Intervention

#### Protocol

A procedure was set up to provide physician faculty with professional development in either one or the other modality in order to approximate a mixed-methods randomized comparative design, with the goal of understanding their perceptions of the two learning modalities. In order to understand their experiences related to the two different educational interventions (PDF text or e-learning module) about providing more effective feedback, participating faculty first received an email that outlined the research study including the purpose and the amount of time estimated to complete the activity. The Qualtrics pre-survey collected the data demographic data (Table 2), and the Qualtrics based internal tool called the Randomizer assigned participants to either the text-only or e-learning educational intervention. This occurred after participant enrollment, with no investigator involvement in assignment. The faculty completed the post-survey, which included a question to determine which educational intervention was utilized, as well as open-ended questions to reflect on the educational intervention (see Table 2). At the end of the post-survey, the final item was a request for volunteers to participate in a focus group to determine the usefulness of the educational intervention as well as a comparison of which modality was favored. The Institutional Review Board determined the study to be exempt.

**Table 2.** Survey Questions

Survey Questions	
Pre-survey	Demographic Questions (age, gender, medical specialty, # years out of training, # years supervising residents)
Post-Survey	To what extent did you find the faculty development useful?*
	To what extent did you find the faculty development changes/will change how you provide feedback to residents?*

*Response scale: 4-point Likert Scale

1 = To a Great Extent

2 = Somewhat

3 = Very Little

4 = Not at All

#### Participants

The main population for this study were the physician faculty of residency programs in a midsized, Midwestern academic teaching center. The sponsoring institution has 8 residency training programs, 2 fellowship training programs, and 143 learners. The faculty were not required to sign up for the study, but could opt in, and by doing so, apply the activity towards faculty development requirements of the accrediting body. All core residency faculty were invited to participate from general surgery (12), pediatrics (8), family medicine (9), and emergency medicine (19) for a total of 48 potential individuals. Of these 48 faculty, 36 (75%) completed the pre-survey, and 29 (60.4%) invitees continued on to also participate in the post-survey. Six (12.5%) self-selected volunteers for the focus group out of this set of participants were from emergency medicine (2), family medicine (1), and surgery (3). Since the self-selected volunteers chose to participate, the focus group was not evenly drawn from both instructional modalities. More of the participants had been randomly assigned to the e-learning modality than the text-based modality via the Qualtrics Randomizer.

In order to study the phenomenon of medical faculty’s experiences with professional development delivered via an e-learning module, a mixed-methods randomized comparative study utilizing thematic analysis was employed.^18^ The utilization of the thematic analysis is to study the subjective experience of those who lived it and gain new insights through rich descriptions.^18,19^ A hermeneutically guided approach seemed appropriate to achieve a robust understanding of the faculty’s perceptions and feelings of eLearning for faculty development.^19^ An independent samples t-test was used to compare the means of two independent groups. This approach was applied because the groups are independent from each other and it was assumed that the normality was reasonably satisfied. The authors acknowledge that analyses were exploratory, findings should be interpreted cautiously, and nonparametric methods were considered. Ultimately, the statistical comparisons applied were exploratory and a power calculation determined the sample size was too small to detect true differences. The thematic analysis of the responses from the focus group interviews were examined to determine if the participants favored the e-learning module or the text-based PDF. Thematic saturation was not formally assessed, as only one focus group with six volunteers was conducted from the pool of 48 invited participants. Additional focus groups may have yielded further themes, so it is acknowledged that full saturation was likely not achieved.

## RESULTS

48 core residency faculty were invited to participate in the study. Of these 48 faculty, 36 (75%) completed the pre-survey, and 29 (60.4%) invitees completed the pre-survey, educational intervention, and the post-survey. Six (12.5%) self-selected volunteers for the focus group. The demographic information describing the participant numbers and characteristics are reported in Table 3.

**Table 3.** Participants Demographic Characteristics

Characteristic	N	Percent
Gender (*N* = 36) Male Female Prefer not to say	20 15 1	55.56% 41.67% 2.78%
Age (*N* = 36) 30-41 42-49 50+ No answer	11 12 12 1	30.56% 33.33% 33.33% 2.78%
Medical Specialty (*N* = 36) Emergency Medicine Family Medicine Pediatrics Surgery	15 5 4 12	41.67% 13.89% 11.11% 33.33%
Years out of residency (*N* = 36) 0-10 Years 11-20 Years 20+ Years No Answer	12 14 7 3	33.33% 38.89% 19.44% 8.33%
Years precepting residents (*N* = 36) 0-10 Years 11-20 Years 20+ Years No Answer	18 10 6 2	50% 27.78% 16.67% 5.56%
Previous faculty development on feedback (*N* = 36) Yes No	28 8	77.78% 22.2%
Faculty Development Modality was PDF or e-Learning Module (*N* = 29) PDF e-Learning Module No Answer	7 21 1	24.14% 72.41% 3.45%

The unequal final group sizes likely resulted from differential attrition following randomization. Thirteen respondents described the e-learning module as useful for the initial learning of the material. Seven respondents described the PDF as a guide to refer back to when utilizing the feedback tool during a feedback session with a resident. The focus group respondents requested to have the PDF laminated and posted in the department and meeting rooms for quick reference (see Table 4).

**Table 4.** Survey Thematic Analysis of Modality Comparison

Survey comments on comparing the e-learning module with the text-based PDF	
e-Learning module was better for learning the material (13 comments)	“Despite the fact that as a physician I feel like I can just read things and remember them, doing the module clearly was better for me.”
	“I really liked [the e-learning module] because there were different points along the way where you'd have questions to test our knowledge to see if we were actually paying attention as opposed to just scrolling through.”
	“There were several opportunities during the module to make sure you understood things.”
PDF is better for follow up reference (7 comments)	The layout was nice. It's like one of those one page cheat sheets unlike the e-learning module where there's several screens to go through if you want a quick and dirty reminder here the e-learning module was not as helpful for that.
	It'd be helpful to put this up on a board in the emergency department. Things that we want to remember, we laminate and then throw up on the boards around [the department].
	The PDF is definitely easy to have access and to use if you're with a resident.
Both modalities have an important role (4 Comments)	So the module for me is more fun, but the PDFs have a role because it's good to have something to pass around in meetings and all.
	We're not saying they're mutually exclusive, right? I'd like both available to me.
	So maybe a combination of both would be helpful. I like modules better than reading this but I totally agree. I could see this being laminated and thrown up on a board in a clinical site that I could glance at if I was having a bad day and couldn't think of how to do this well

Performance and impact are indicated by the focus group interview analysis, which revealed that the faculty had plans for utilizing the text-based PDF in the trauma conference room, for posting it on the emergency department notice board, and made further requests to require the educational intervention as part of faculty development at orientation and ongoing sessions (see Table 5).

**Table 5.** Focus Group Outcome Evaluation

Focus Group Comments on Utilizing the Educational Intervention	
Text-Based PDF	This came up in our trauma division meeting, and I was thinking too that if I have a copy, I can put it up in my trauma conference room.
	It sends a message because, you know, we have morning sit down rounds for the residents, so every day seeing this, and my colleagues seeing this, and my trauma colleagues seeing this, it is a way to spread this.
	I suggest laminating them and putting them up in our trauma conference room, and then at the end of our trauma division meetings, which the 3 of us get together every week, we could kind of mention this. And that way we can actually discuss the evaluations ourselves and that way, you'll get a more uniform product and perhaps a more accurate 1 out of the trauma rotation.
e-Learning Module	It would be really helpful if all of the faculty are on the same page, so it might be that modules are meant to be done on your own time or when you're sitting at home, that maybe the setup for education would be everybody do the short 5 or 10 minute module then while we're sitting in the room together, we can talk about it and really get everybody on the same page.
	Being able to require faculty to do it [the e-Learning module] before we have our semi-annual meeting would probably be very helpful … So I think this helps to get everybody to standardize to some extent.
	After doing this [e-Learning module]… it sounds like the trauma folks are talking about maybe, um, doing a mini session about milestones.

## DISCUSSION

In this study, 36 of the 48 invited faculty completed the pre-survey, 29 invited faculty completed the pre-survey, educational intervention, and post-survey, and 6 invited faculty participated in a focus group. Quantitatively, 13 respondents preferred the e-learning module for initial learning, while 7 preferred the PDF for reference during resident feedback sessions. Future use preferences were evenly distributed between modalities. Qualitatively, thematic analysis revealed the e-learning module better provided foundational knowledge, while the text-based modality was valued reference during in-person feedback settings. Both were seen as complementary for faculty development. This study revealed the usefulness for providing resources in more than one modality when delivering faculty development. No statistically significant difference between the two modalities was detected in how faculty reported plans to provide feedback to residents. Given the small and unequal group sizes and limited statistical power, this finding should not be interpreted as evidence of equivalence between the modalities. This may indicate that the information and education provided in both of the modalities was effectively mirrored (and, to ensure equitable training opportunities, may also indicate that the study was successfully focused on the comparison of the text-based PDF versus e-learning module delivery system without an uneven distribution of effects). Alternative explanations beyond the statistical results are the participants’ prior faculty development experience (78% had previous training), personal preference and familiarity with electronic versus printed formats of educational delivery, or the short timeframe to apply behavioral change. While the survey indicated that some faculty felt the e-learning educational activity was less than helpful, the focus group faculty later reported that the activity started a conversation about the importance of feedback; while it is too early to make any reasonable conclusions about the effects of this online training as compared to the PDF, focus group participants indicated that preliminary discussions among faculty suggested increased attention to feedback practices.

This study has its limitations including small sample size, uneven group allocation, specialty diversity, and generalizability since the study involved a single institution setting and was a mixed-methods randomized comparative exploration of a professional development intervention, not a study of specific effects based on a randomized, controlled research design. Due to the nature of the study splitting the participants into two groups, the smaller initial sample size limited the number of results of each survey. 36 faculty completed the pre-survey, however, only 29 completed the post-survey. Of the 29 faculty, 7 indicated they received the text-only PDF and 21 indicated they received the e-learning module (and one chose not to answer). The self-selection for the focus group may have reflected the views of those more interested in the topic, potentially narrowing the range of perspectives captured, and the findings rely on self-reported perceptions rather than objective educational outcomes. Finally, as noted, this mixed-methods randomized comparative study - conducted by the lead author as a researcher who also serves as an instructor of education and accreditation manager - was designed to understand the perceptions of faculty on e-learning as the delivery method for faculty development.

## CONCLUSION

With the swift move towards e-learning prompted by medical advancement and the 2020 COVID-19 pandemic,^1,5,20^ this study aimed to discover the usefulness of e-learning in medical faculty development. While this study did not determine if either modality was effective for providing faculty development in a generalizable manner, the research did uncover a complementary set of ways to deliver education to medical faculty - both in traditional textual formats and through e-learning modules - and provide them with multiple tools for reference and retention. The implications of this study suggest that faculty development interventions could utilize e-learning modalities for initial engagement and foundation building, while text-based modalities may function better for ongoing reference and in-person meetings and discussions. Next steps include evaluating usage patterns and feedback technique retention over 6-12 months. Future research could include testing a combined modality approach to faculty development, as well as measuring causal effects which could determine if the content of the modalities affected the perceptions of e-learning usefulness.

### Conflict of Interest

None

### Financial Support

None

### IRB Statement

The Central Michigan University Office of Research Compliance determined this project to be exempt/non-human subjects research under 45 CFR 46.102(l)

### Reflexivity

The lead author acknowledges their dual role in conducting and analyzing this study and recognizes the potential for bias. To mitigate potential bias, co-authors external to the host institution contributed to data interpretation and manuscript review.

### CRediT Contributor Roles Taxonomy

Bethany Figg: Conceptualization, Methodology, Formal Analysis, Investigation, Data Curation, Writing – Original Draft, Visualization

Troy Hicks: Methodology, Supervision, Validation, Writing – Review & Editing

Sally Santen: Conceptualization, Methodology, Supervision, Writing – Review & Editing

Clara Bihn: Validation, Writing – Review & Editing

Steve Vance: Data Curation, Software, Visualization

Mary Jo Wagner: Supervision, Validation, Writing – Review & Editing
